# Low-damage direct patterning of silicon oxide mask by mechanical processing

**DOI:** 10.1186/1556-276X-9-269

**Published:** 2014-05-29

**Authors:** Shojiro Miyake, Shohei Yamazaki

**Affiliations:** 1 Department of Innovative System Engineering, Nippon Institute of Technology, 4-1 Gakuendai, Miyashiro-machi, Saitama 345-8501, Japan

**Keywords:** Nanofabrication, Mechanochemical processed layer, Atomic force microscopy, Natural oxide layer

## Abstract

To realize the nanofabrication of silicon surfaces using atomic force microscopy (AFM), we investigated the etching of mechanically processed oxide masks using potassium hydroxide (KOH) solution. The dependence of the KOH solution etching rate on the load and scanning density of the mechanical pre-processing was evaluated. Particular load ranges were found to increase the etching rate, and the silicon etching rate also increased with removal of the natural oxide layer by diamond tip sliding. In contrast, the local oxide pattern formed (due to mechanochemical reaction of the silicon) by tip sliding at higher load was found to have higher etching resistance than that of unprocessed areas. The profile changes caused by the etching of the mechanically pre-processed areas with the KOH solution were also investigated. First, protuberances were processed by diamond tip sliding at lower and higher stresses than that of the shearing strength. Mechanical processing at low load and scanning density to remove the natural oxide layer was then performed. The KOH solution selectively etched the low load and scanning density processed area first and then etched the unprocessed silicon area. In contrast, the protuberances pre-processed at higher load were hardly etched. The etching resistance of plastic deformed layers was decreased, and their etching rate was increased because of surface damage induced by the pre-processing. These results show that etching depth can be controlled by controlling the etching time through natural oxide layer removal and mechanochemical oxide layer formation. These oxide layer removal and formation processes can be exploited to realize low-damage mask patterns.

## Background

In nanotechnology, nanoelectric devices and nanomachines can be manufactured by manipulating atoms and molecules [1]. Nanofabrication is one of the most important aspects in the development of nanotechnology. Scanning probe microscopy (SPM) is useful for the nanofabrication of nanometer-scale engineering materials and devices [2] and can be used to realize atomic-scale fabrication. Various attempts have also been made to use SPM techniques for the local modification of surfaces [2-4]. In particular, the local oxidation technique is expected to allow the fabrication of electric devices on the nanometer scale [5-7]. The oxide layers formed by this technique can function as a mask during the etching step or can be used directly as an insulating barrier [7]. In this method, oxidizing agents contained in surface-adsorbed water drift across the silicon oxide layer under the influence of a high electric field, which is produced by application of a voltage to the SPM probe.

Mechanical processing methods that transcribe a tool locus can produce three-dimensional nanoprofiles with high precision by exploiting the tribological properties of the tool geometry and workpiece [8,9]. If profile processing using mechanical action can be achieved at nanometer scales, the degrees of freedom of the materials that can be used and the range of profiles and sizes of the objects that can be processed will be greatly increased [10-13]. Therefore, the applications of nanofabrication can be expected to be significantly extended through such novel processes [8-13].

Meanwhile, processing methods combining both mechanical and chemical actions have been widely used to machine high-quality surfaces with high precision [14]. Mechanochemical polishing (MCP) uses mechanical energy to activate chemical reactions and structural changes. The processing of highly flat surfaces with few defects has been made possible by this method. Recently, the so-called chemical-mechanical polishing (CMP) has been applied to the fine processing of electronic devices [15]. Further, a complex chemical grinding approach that combines chemical KOH solution etching and mechanical action has been studied [16]. These combined mechanochemical processing methods can achieve high-precision and low-damage machining, simply by using mechanical action to promote reactions with atmospheric gas and surface adsorption layers.

Atomic force microscopy (AFM) is a useful technique for mechanical nanofabrication [8-10]. Mechanical friction methods have been used for the fabrication of silicon nanostructures on H-passivated Si (100) substrate [17], and the so-called maskless [18,19] or friction-induced nanofabrication [20-22] has also been proposed. However, the mask patterns formed by these methods are mechanically produced at higher load and stress, damaging the mask surfaces and creating an oxidation layer that decreases the etching rate achieved with KOH solution. As a result, these damages remain on the processed surfaces [18-22].

In our previous study, we proposed a lower damage direct patterning of oxide layers by mechanical processing. Sliding of an AFM diamond tip on a silicon surface forms protuberances under ambient conditions [23-25]. Proper mechanical action without plastic deformation by a sliding diamond tip on a silicon surface results in local mechanochemical oxidation with low damage [23-26]. The resulting oxide masks can be used for pattern transfer during selective wet etching processes [24-28].

Subsequently, by changing the diamond tip sliding scanning density, we realized the control of the etching rate of a silicon surface by KOH solution. We also evaluated the dependence of etching depth on KOH solution etching time [26]. An approach combining mechanical and electrical processes, such as an AFM technique that simultaneously uses a mechanical load and bias voltage, could be developed in the future. Reports on electrical and mechanical nanoprocessing have indicated that this complex approach can produce more electrically resistant layers [29].

In this study, we attempted to fabricate a nanometer-scale etching mask pattern with low damage and evaluate the chemical resistance properties of the mechanically processed areas. First, we removed the natural oxide layer by diamond tip sliding at low load and then increased the etching rate with KOH solution. Then, at higher load, we formed an etching resistance layer using mechanochemical oxidation. We fabricated protuberances with and without plastic deformation by mechanical processing. Finally, the surfaces were processed at low load and scanning density to remove the natural oxide layer. The dependence of the KOH solution etching depth of these processed areas on etching time was also investigated.

## Methods

The specimens were n-type Si (100) wafers. The samples were exposed in a clean atmosphere to allow their surfaces to become covered with a natural oxide layer less than 2 nm thick. First, mechanical processing was performed using diamond tip sliding with an AFM under atmospheric conditions at room temperature and humidity ranging between 50% and 80%.

### Dependence of KOH solution etching on load and scan density of mechanical pre-processing

**Figure 1:**
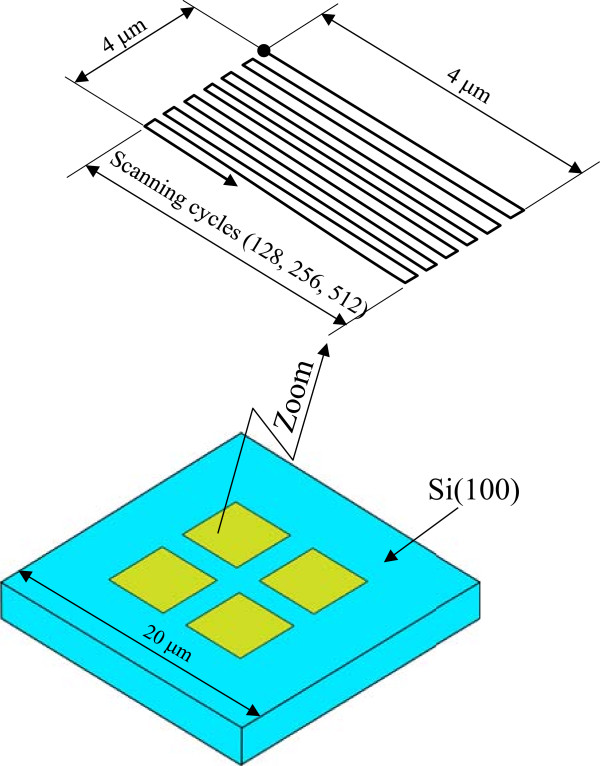
**Mechanical pre**-**processing method.**

We clarified the conditions under which the etching rate increased after mechanical pre-processing due to the removal of the natural oxide layer. To evaluate the dependence of the KOH solution etching of the mechanically pre-processed area on the applied load and scanning density, diamond tips were directly slid on the Si (100) using the AFM, and square areas were processed as shown in Figure 1. The changes in the profiles of the processed areas were observed for a light applied load and an expanding scanning range. The Si (100) specimens were driven with the diamond tip at various load conditions. Scanning was performed 128, 256, and 512 times on a 4 × 4 μm^2^ area. To realize protuberance formation and plastic deformation, 100 ± 10 nm radius diamond tips were selected [23].

KOH solution etching of the pre-processed silicon substrate with 10 wt% KOH solution at 20°C ± 3°C was performed on the AFM apparatus. After etching, the specimen was washed with distilled water, and the profile changes caused by the etching were then evaluated at the same positions using the same diamond tip as the processing tool.

### Dependence of additional KOH solution etching on etching time

**Figure 2:**
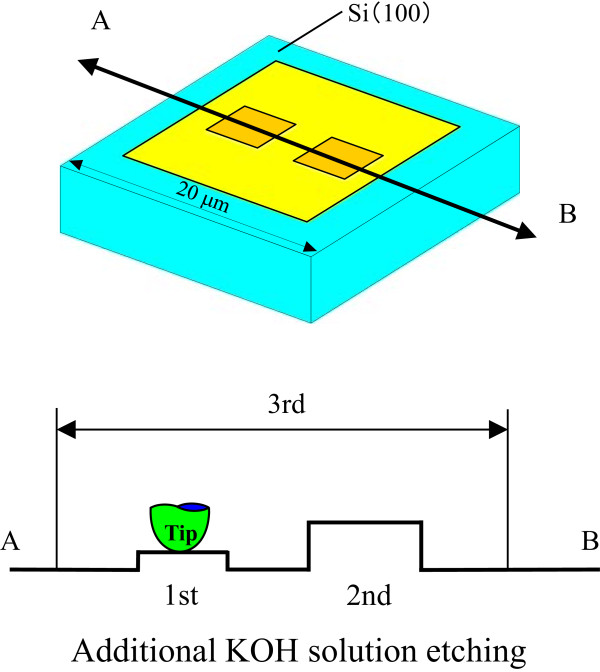
Mechanical and additional pre-processing.

Three types of mechanical pre-processing were performed, as shown in Figure 2. For the first and second, the silicon surfaces were processed at 10- and 40-μN load at 1 × 1 μm^2^, respectively. Diamond tip sliding at 10-μN load and 256 scanning number produced protuberance. At 40-μN load, the processed area protuberated, and plastic deformation began [27,28]. Under these load conditions, the processed layers prevented KOH solution etching. For the third type of pre-processing, the sample was slid at 1.5-μN load and 256 scans in a 5 × 5 μm^2^ area. Finally, the processed samples were etched with 10 wt% KOH solution at 20°C ± 3°C for 10, 25, 30, and 40 min. Changes in the topography of the sample during the etching process were observed by tip scanning at less than 0.3 μN over an area of 15 × 15 μm^2^.

## Results and discussion

### Dependence of KOH solution etching on mechanical pre-processing owing to the removal of the natural oxide layer

**Figure 3:**
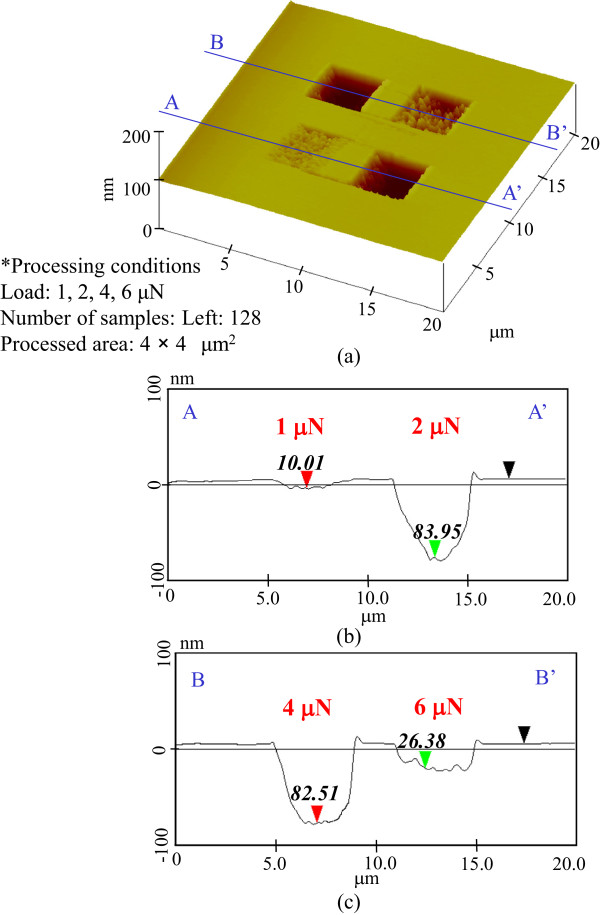
**Etching profile for 128-scan pre-processing. (a)** Surface profile. **(b)** Section profile (1 and 2 μN). **(c)** Section profile (4 and 6 μN).

**Figure 4:**
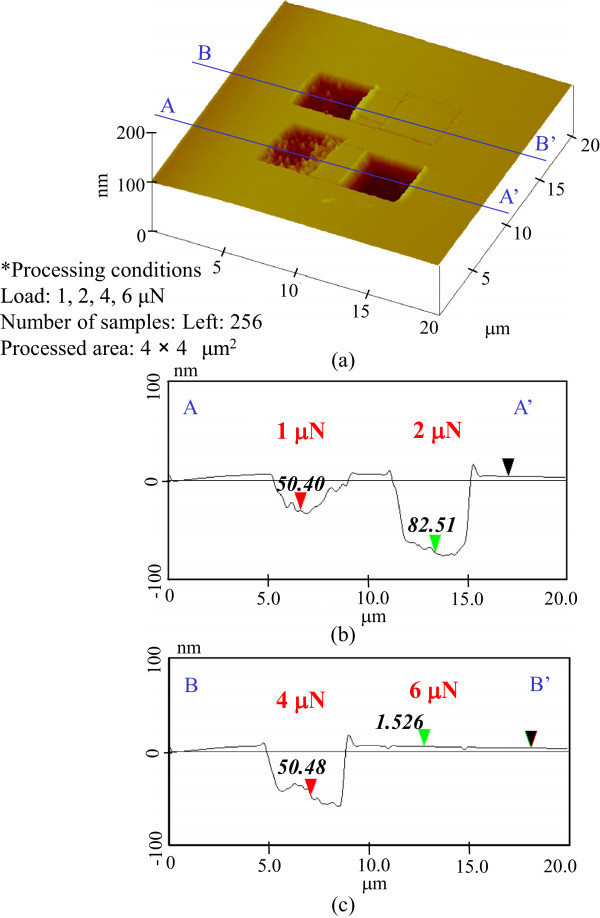
**Etching profile for 256-scan pre-processing. (a)** Surface profile. **(b)** Section profile (1 and 2 μN). **(c)** Section profile (4 and 6 μN).

**Figure 5:**
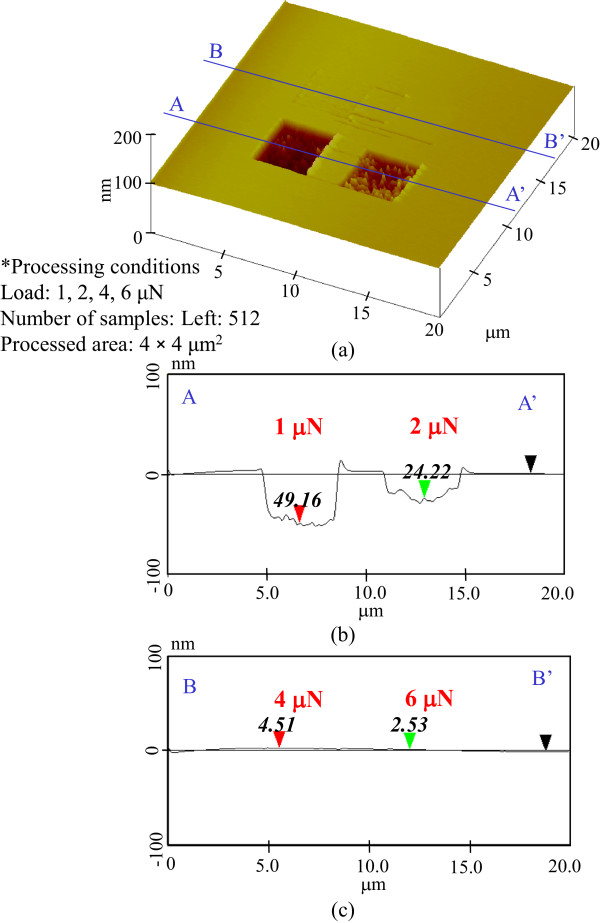
**Etching profile for 512-scan pre-processing. (a)** Surface profile. **(b)** Section profile (1 and 2 μN). **(c)** Section profile (4 and 6 μN).

**Figure 6:**
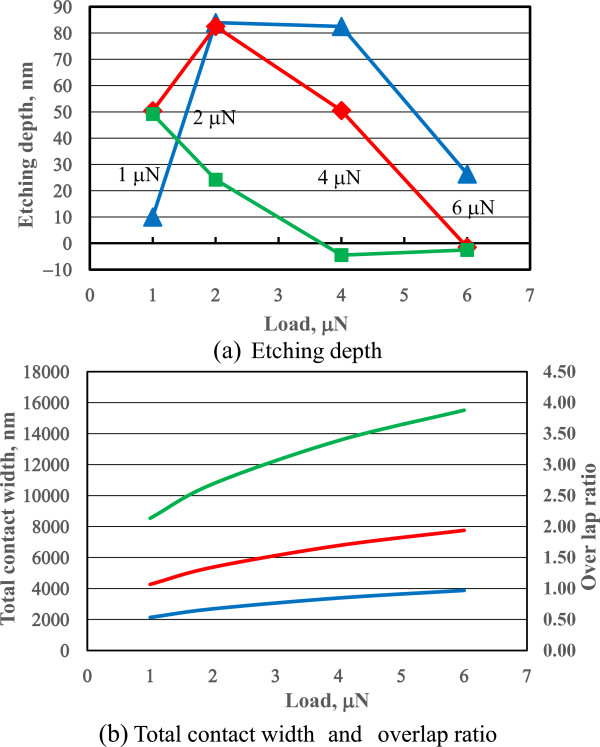
Dependence of etching depth (a) and overlap ratio (b) on load and scanning number of pre-mechanical processing.

To clarify the mechanism responsible for the increase in the etching rate on the removal of the natural oxide layer, the mechanical pre-processing was performed at 1-, 2-, 4-, and 6-μN load. The dependence of the etching profile on the pre-processing load at 128 scans is shown in Figure 3. The etching depths of the samples pre-processed at 1- and 2-μN load were 10 and 84 nm, respectively. At 4-μN load, the etching depth was saturated at 83 nm. However, the etching depth decreased to 26.3 nm at 6-μN load. Thus, the greatest etching depths were obtained at the 2- and 4-μN-load pre-processed areas.Furthermore, for 256 scans, the etching depths were 50 nm at 1-μN load, 83 nm at 2-μN load, 50 nm at 4-μN load, and 0 nm at 6-μN load, as shown in Figure 4. The largest etching depth, 83 nm, was obtained in the areas pre-processed at 2-μN load. Figure 5 shows the etching profiles of pre-processed areas scanned 512 times. The greatest etching depth obtained after 512 scans was 50 nm at the lowest load of 1 μN.Figure 6a shows the dependence of etching depth on the pre-processed load. Under these conditions, the unprocessed areas were negligibly etched. A load range within which the etching depth increased existed for every scanning number, and these load ranges tended to increase as the number of scans decreased. The contact pressure and contact diameter were evaluated using the Hertzian equation. At 1 and 6 μN, the contact pressures were 6.9 and 12.5 GPa, respectively.The scanning density decreased with the scanning cycle number. The total contact sliding width can be evaluated from the product of the contact diameter and scan number. Then, to evaluate the overlap ratio, the total contact width is divided by the scanning width. For example, at 6-μN load, the Hertzian contact diameter is nearly 30.3 nm; therefore, the total contact width for 128 scans was 30.3 × 128 nm and the overlap ratio was nearly 0.97, as shown in Figure 6b. In this case, the total contact width was smaller than the scanning width. The natural oxide layer formed on the Si surface was removed at low scan number conditions; overlap of the sliding contact area appeared to produce an etching-resistant layer.

**Figure 7:**
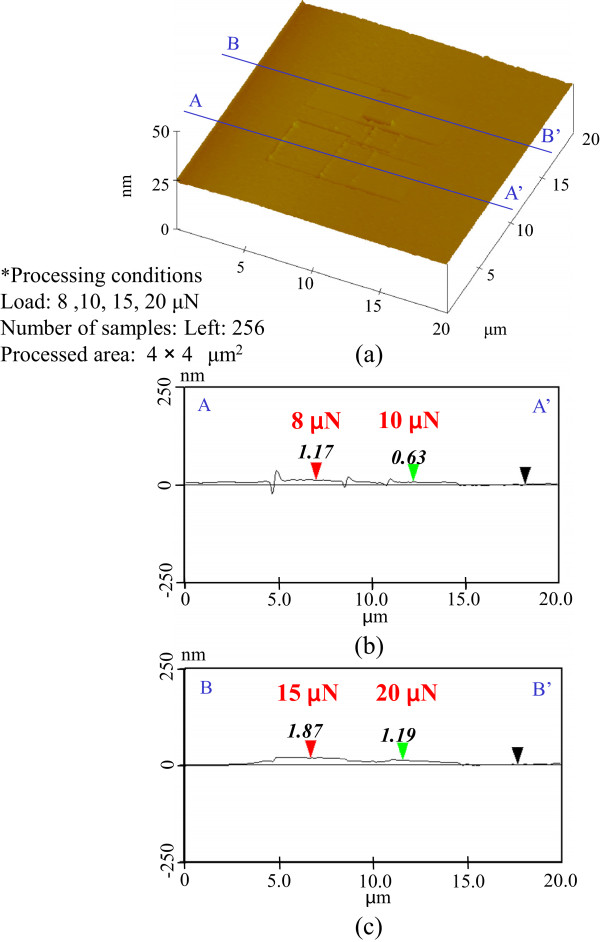
**Etching profile processed at higher load with 256 scans. (a)** Surface profile. **(b)** Section profile (8 and 10 μN). **(c)** Section profile (15 and 20 μN).

**Figure 8:**
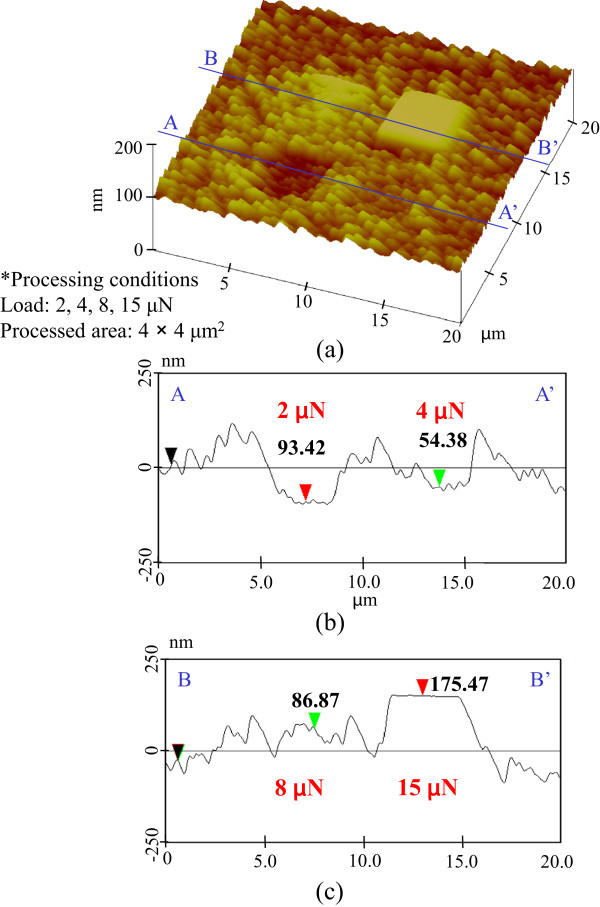
**Etching profile of pre-processed area at higher load. (a)** Surface profile. **(b)** Section profile (2 and 4 μN). **(c)** Section profile (8 and 15 μN).

Owing to the removal of the natural oxide layer, 512 scans at 1-μN load also increased the etching rate. Processing at higher loads of 4 and 6 μN increased the amount of mechanochemical oxidation owing to the high density of the scanning and thus decreased the etching depth. At 512 scans, the total contact width was larger than the scanning width, so the contact area overlapped. Pre-processing at low load and scanning density efficiently removed the natural oxide layer by mechanical action while also mechanochemically generating a thin oxide layer because of the sliding overlap.To clarify the etch properties of pre-processed areas at higher load, the etching profiles obtained at 8-, 10-, 15-, and 20-μN load after 256 scans were evaluated as shown in Figure 7. In these cases, etching grooves could not be detected in any of the processed areas. The heights of all of the processed areas were slightly greater than those of the unprocessed areas. Thus, the effect of any increases in etching rate resulting from the removal of the natural oxide layer could not be obtained. This is conceivable because mechanochemical oxidization increases at higher load, resulting in improved resistance towards etching with KOH solution.To compare the resistances of the natural oxide layer and the mechanochemically generated oxide layer to etching, we extended the etching time by 5 min. Figure 8 shows the etching profiles of pre-processed areas at 2-, 4-, 8-, and 15-μN loads. In this case, etching grooves were observed in pre-processed areas at 2 and 4 μN. In contrast, the heights of the pre-processed areas at 8 and 15 μN were higher than that of the unprocessed area. This is conceivable because the areas pre-processed at 8- and 15-μN load had better etching resistance towards KOH solution than that of the natural oxide layer. The etched silicon surfaces were very rough because the etching rate changed over different features on the surface, such as areas of damage, oxide, and adsorbates.

Therefore, with 256 scanning cycles, mechanical pre-processing at a load of 1 to 4 μN was effective in increasing the etching rate. Over 8-μN load, mechanical pre-processing was effective in forming an etch-resistant layer on the Si surface.

To clarify the mechanism of the mechanical removal and formation of this etch-resistant layer, the surface contact stress was evaluated using the boundary element method [27]. The dependences of the maximum principal and shear stresses on load were estimated for 100-nm-radius diamond tips. The 1- to 4-μN-load range corresponds to a contact pressure of 6.9 and 10.9 GPa. Therefore, it can be concluded that this contact pressure range is suitable for the removal of the natural oxide layer on a silicon surface at low-density scanning.

Silicon fractures under tensile stress at a certain load. In maximum tensile stress areas, silicon bond breakage appears to stem from tensile stress caused by diamond tip friction [27]. Therefore, the reaction of silicon may take place at the rear edge of the sliding contact area where the elongation stress is the highest. At loads of over 8 μN, protuberance height increased rapidly at 13.8-GPa contact pressure and 1.8-GPa tensile stress. Therefore, this protuberance-related phenomenon occurred through a mechanochemical reaction where adsorbates, such as water and oxygen, reacted with the silicon. The local destruction of interatomic bonds seems to increase at over 6 μN because of the concentrated stress and reaction of the newly formed surface with surrounding materials. This boundary load that increases and decreases the etching depth is nearly 6 μN. At this load, the contact pressure and tensile stress are 12.5 and 1.5 GPa, respectively.

### Additional KOH solution etching of processed protuberances with and without plastic deformation

As mechanical pre-processing, protuberances with and without plastic deformation were processed at 10- and 40-μN loads. It was found that less surface damage occurred than that due to plastic deformation during the nanoprocessing on Si. The shear stress was evaluated to estimate the plastic deformation of the silicon, and the effect of the evaluated contact stress on protuberance height and groove depth was studied [27,28].

**Figure 9:**
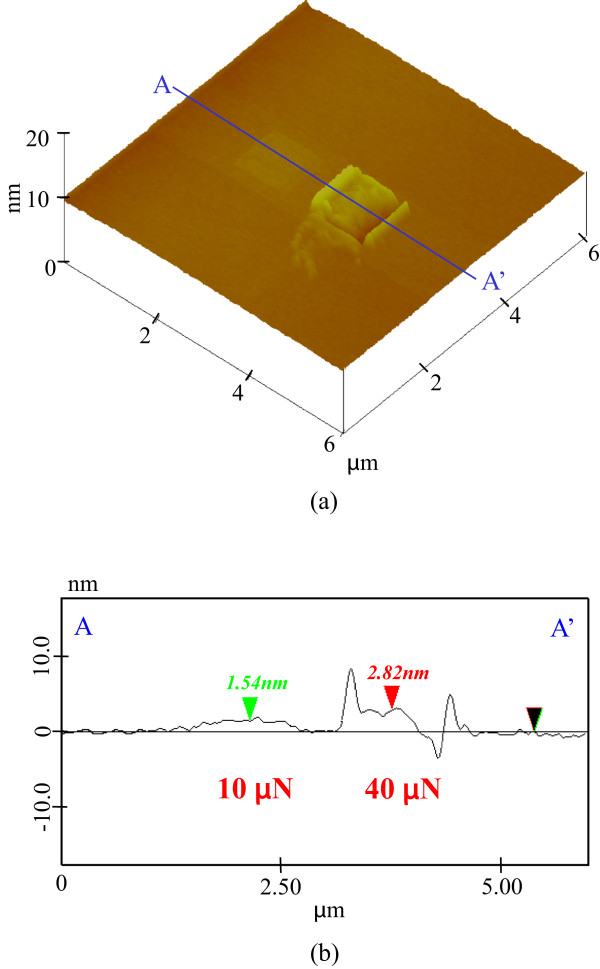
**Profile of the Si (100) surface processed by diamond tip sliding. (a)** Surface profile. **(b)** Section profile (10 and 40 μN).

The presence of a mechanochemical local oxide layer prevented KOH solution etching. Protuberance heights increased until the tensile stress reached 4.5 GPa and then decreased with load. At this peak height, the maximum shear stress attained was more than 8 GPa. This suggests that mechanochemical processing using a 100-nm-radius diamond tip is load dependent when the shear stress exceeds the strength of silicon, inducing a plastic deformation of several nanometers. Additional KOH solution etching was performed on the processed silicon to evaluate the chemical properties of the processed area. The topography and cross-sectional profiles of a silicon sample pre-processed with a 100-nm-radius diamond tip at loads of 10 and 40 μN were obtained by scanning at 1.5 μN over an area of 6 × 6 μm^2^ as shown in Figure 9. At 10-μN load, a 1.5-nm-high protuberance was mechanochemically generated by the sliding of the diamond tip. In contrast, at 40 μN, the height of the protuberance reached 3 nm as shown in Figure 2, while plastic deformation produced a groove at the end of the scanning area. The natural oxide layer was removed under the 1.5-μN load at 6 × 6 μm^2^ scanning area and 256 scanning cycles. At nearly 10-μN load, the 100-nm-radius tip produced protuberances of nearly 1.5 nm through silicon oxidation. However, the maximum shear stress increased beyond the yield criterion at nearly 40-μN load, resulting in silicon plastic deformation and a subsequent change in profile. In this condition, the height of the processed area was as much as 3 nm higher than that of the area processed at 10-μN load, and surface damages such as dislocations were increased in number.

To understand the dependence of the relative etching depth on etching time, the pre-processed and unprocessed areas were etched with KOH solution for 10, 15, 20, 25, 30, and 40 min. No significant change in the topography of the surface was observed even after 10- and 15-min etching. The heights of the protuberances were slightly increased to 2.3 and 3.4 nm at 10 and 40 μN, respectively.

**Figure 10:**
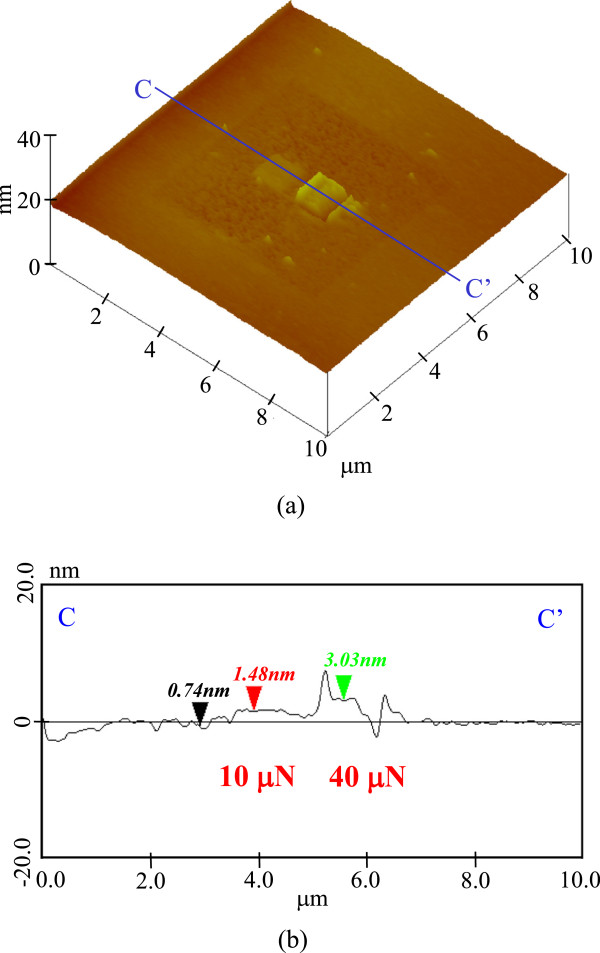
**Etching profile of processed parts after 20 min. (a)** Surface profile. **(b)** Section profile (10 and 40 μN).

**Figure 11:**
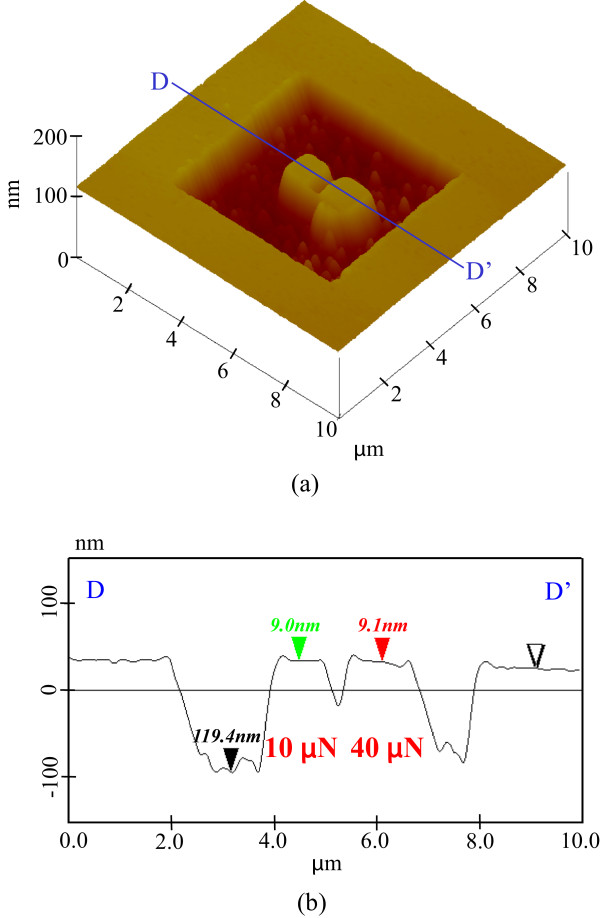
**Etching profile of processed parts after 25 min. (a)** Surface profile. **(b)** Section profile (10 and 40 μN).

**Figure 12:**
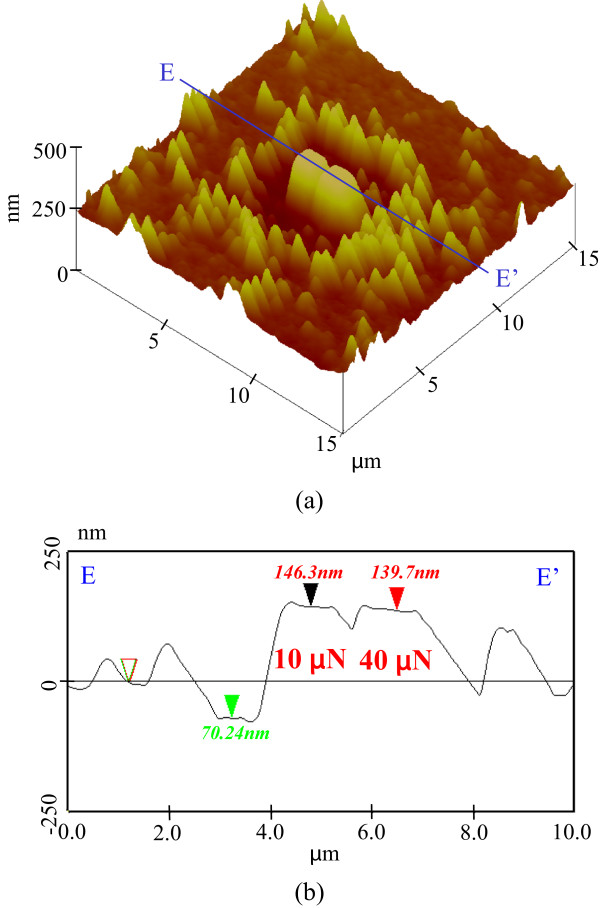
**Etching profile of processed parts after 30 min. (a)** Surface profile. **(b)** Section profile (10 and 40 μN).

**Figure 13:**
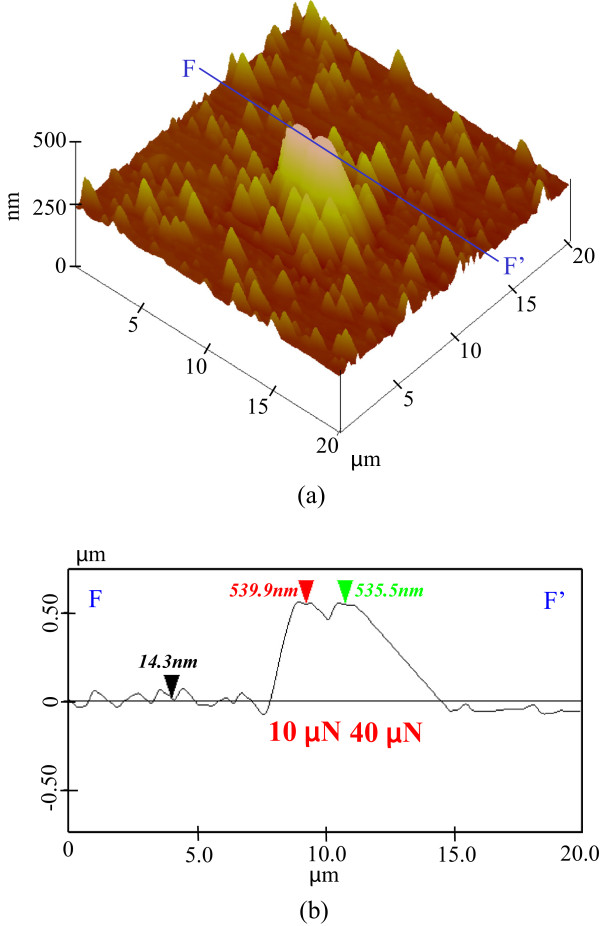
**Etching profile of processed parts after 40 min. (a)** Surface profile. **(b)** Section profile.

**Figure 14:**
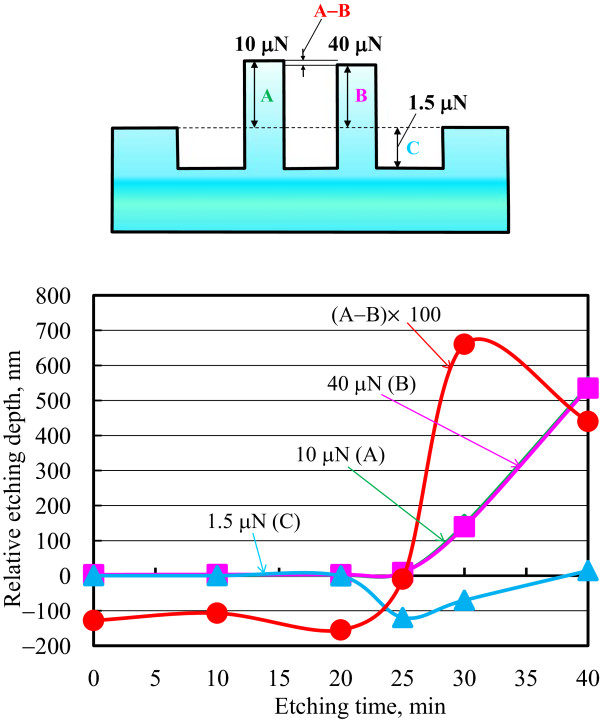
Dependence of relative etching depth on etching time at different loads.

Figure 10 shows the topography and cross-sectional profiles of the processed surface after 20-min KOH etching. The square groove of the 6 × 6 μm^2^ area processed at 1.5-μN load was slightly etched. Although the depth of this groove was 1 nm or less, the roughness of the processed surface was slightly increased. Meanwhile, the area pre-processed at 10 and 40 μN was not etched.Figure 11 shows the etching profile of pre-processed areas after 25 min. The etching depth of the area pre-processed at 1.5-μN load was significantly increased to more than 110 nm. This rapid increase in etching depth was due to the removal of the natural oxide layer by the low-load pre-processing. In contrast, the changes in the profiles of the areas pre-processed at 10 and 40 μN, and the basal plane of the unprocessed areas, were very small. The natural oxide layer worked as an etching mask at 25 min. While the heights of the pre-processed areas were exactly the same as those before etching, the area pre-processed at 40-μN load was enlarged by the plastic deformation.Figure 12 shows the topography and cross-sectional profiles of the pre-processed areas after 30-min etching. The etching also advanced in the unprocessed area. The etching depth of the area processed at 1.5 μN progressively increased to 210 nm, while that of the unprocessed area increased to 140 nm. This implied that only the high-loaded processed area was not etched because of the mechanochemical oxide layer. The height obtained at 10-μN load was slightly higher than that at 40-μN load.Figure 13 shows the etching profile of pre-processed areas after 40-min etching. The etching depths of both the low-load processed and unprocessed areas were approximately 530 nm. In contrast, the areas processed at high loads of 10 and 40 μN were not etched. This experimentally confirmed that high-loaded processed protuberate areas show superior etching resistance towards KOH solution due to formation of a high-density oxide layer.Figure 14 shows the dependence of relative etching depth on KOH solution etching time. The standard plane is the unprocessed area. The plane heights of the areas pre-processed at 10- and 40-μN load from the standard plane are denoted as A and B. The corresponding height of the area pre-processed at 1.5-μN load is C. Between 10 and 20 min, there was little change in the topography of each area. From 25- to 30-min etching, it was observed that the etched depths significantly increased in the 1.5-μN-load pre-processed area. However, etching was hardly observed in the 10- and 40-μN-load pre-processed areas. Etching of the unprocessed area was hardly observed until 25 min. After 30-min etching, the unprocessed area was progressively etched owing to the removal of the natural oxide layer.

From 35 to 40 min, the etching depths of both the unprocessed and 1.5-μN-load pre-processed areas were larger than those of the areas processed at higher load. The area mechanically pre-processed at higher load exhibited resistance to etching owing to mechanochemical oxidation layer formation.

**Figure 15:**
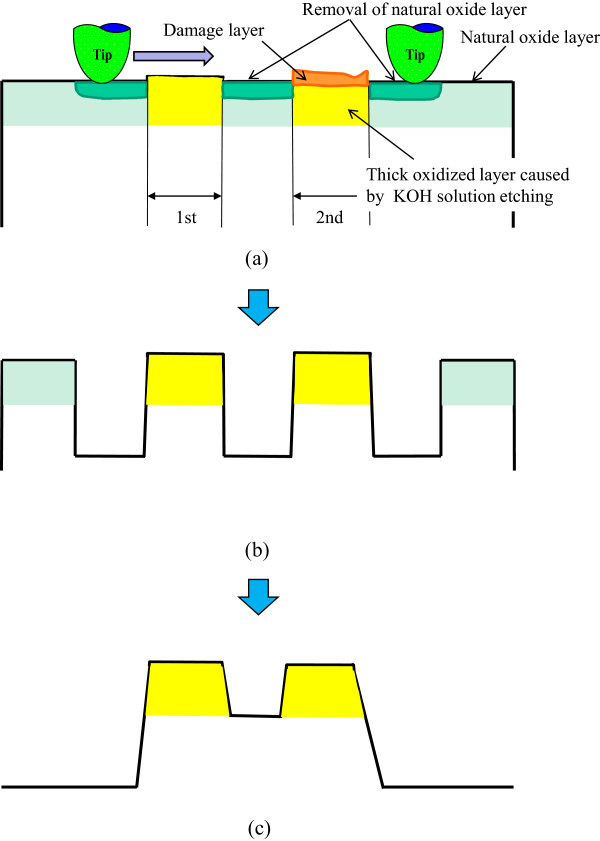
**Model of the increasing and decreasing of etching rate. (a)** Change to surface profile by mechanical processing. **(b)** Change to surface profile by KOH solution etching (25 min). **(c)** Change to surface profile by KOH solution etching (40 min).

The difference between the heights of the 10-μN uniform protuberance and the 40-μN protuberance with plastic deformation can be evaluated as A-B. Without etching, the height of the area pre-processed at 10-μN load was lower than that at 40 μN. When the KOH solution etching time was increased, A-B was nearly 3 nm until 20 min. The heights of the areas were similar in value at 25 min. In contrast, at 30- and 35-min etching time, the height of the 10-μN load area was higher than that at 40 μN. These results show that the etching rate of the area pre-processed at 40-μN load was larger than that at 10 μN. This is deduced to be because the area pre-processed with plastic deformation at 40-μN load was more easily etched due to damage compared with the uniform protuberance pre-processed at 10 μN.Figure 15 shows a model of etching depth dependence on KOH solution etching time for pre-processed areas. As shown in Figure 15b, with an increase of etching time, by the removal of the natural oxide layer, the 1.5-μN-load pre-processed area was etched at first. The etching rate increased with KOH solution etching time under processing at low load and scanning density.However, as shown in Figure 15c, the two areas processed at higher load and scan density were not etched because of their thick oxide layers. These thick oxide layers, which were mechanochemically formed on the areas processed at higher load, prevented the KOH solution etching and thereby decreased the etching rate. From these results, the etching rate is controllable by the removal of the natural oxide layer and direct oxidation by mechanical action. Grooves with various depths can be obtained using this etching rate control.

## Conclusions

To realize the nanofabrication of a Si substrate, the etching depths obtained with KOH solution were controlled using mechanical pre-processing under various loads and scanning density conditions. Removal and formation of the oxide etching mask was performed on silicon surfaces using atomic force microscopy.

Areas mechanically pre-processed at 1- to 4-μN load exhibited an increased KOH solution etching rate due to the removal of the natural oxide layer by the mechanical action. The dependence of etching depth on pre-processing load and scanning density was clarified. At every scanning density, there were certain load ranges within which the etching depth increased. In contrast, protuberances with a thick oxide layer produced by mechanical pre-processing at higher load suppressed etching. This mechanochemical oxide layer had superior etching resistance to that of the natural oxide layer.

Protuberances were processed on the Si surfaces under stress conditions both lower and higher than that where plastic deformation occurs. These processed areas were hardly etched by the KOH solution. For protuberances with plastic deformation, the damaged layers were more easily etched than those without plastic deformation. Protuberance formation without plastic deformation by mechanical pre-processing can realize less damaged mask patterning. Additionally, areas at pre-processed low load and scanning density were easily etched. This implies that the various profiles obtained were possibly fabricated by the changing load and scanning density of the mechanical pre-processing and by additional KOH solution etching. With the removal of the natural oxide layer and formation of a mechanochemical oxide layer without plastic deformation, the etching depth can be controlled by changing the etching time. This therefore allows us to fabricate low-damage grooves of various depths.

## Competing interests

The authors declare that they have no competing interests.

## Authors’ contributions

SM carried out the nanofabrication studies, participated in the nanoprocessing using atomic force microscopy, and drafted the manuscript. SY carried out and evaluated the Si nanoprocessing experiment and helped to draft the manuscript. All authors read and approved the final manuscript.
